# Sialidase facilitates *Porphyromonas gingivalis* immune evasion by reducing M1 polarization, antigen presentation, and phagocytosis of infected macrophages

**DOI:** 10.3389/fcimb.2023.1173899

**Published:** 2023-05-30

**Authors:** Xiaomiao Fan, Shaowen Zheng, Chen Chen, Li Lin, Hongyan Wang, Yuqin Shen, Yaping Pan, Chen Li

**Affiliations:** ^1^ Department of Periodontology, School and Hospital of Stomatology, China Medical University, Liaoning Provincial Key Laboratory of Oral Disease, Shenyang, Liaoning, China; ^2^ Department of Periodontics, Affiliated Stomatology Hospital of Guangzhou Medical University, Guangdong Engineering Research Center of Oral Restoration and Reconstruction, Guangzhou Key Laboratory of Basic and Applied Research of Oral Regenerative Medicine, Guangzhou, Guangdong, China

**Keywords:** periodontitis, *Porphyromonas gingivalis*, sialidase, macrophage, polarization

## Abstract

**Background:**

*Porphyromonas gingivalis* (*P. gingivalis*), a major pathogen of periodontitis, can evade host immune defenses. Previously, we found that *P. gingivalis* W83 sialidase gene mutant strain (ΔPG0352) was more easily cleared by macrophages. The aims of this study were to investigate the effects of sialidase in *P. gingivalis* on the polarization, antigen presentation, and phagocytosis of infected macrophages and to clarify the mechanism of *P. gingivalis* immune evasion.

**Methods:**

Human monocytes U937 were differentiated to macrophages and infected with *P. gingivalis* W83, ΔPG0352, comΔPG0352, and *Escherichia coli* (*E. coli*). The phagocytosis of macrophages was observed by transmission electron microscopy and flow cytometry. ELISA or Griess reaction were used to examine the levels of interleukin-12 (IL-12), inducible nitric oxide synthase (iNOS) and interleukin-10 (IL-10), and the expressions of CD68, CD80 and CD206 were determined by flow cytometry. The expression of major histocompatibility complex-II (MHC-II) was detected by immunofluorescence. A rat periodontitis model was established to determine the M1 and M2 polarization of macrophages.

**Results:**

Compare with *P. gingivalis* W83, ΔPG0352 increased the levels of IL-12, iNOS, CD80, and MHC-II and inhibited the levels of IL-10 and CD206. Macrophages phagocytosed 75.4% of ΔPG0352 and 59.5% of *P. gingivalis* W83. In the rat periodontitis model, the levels of M1 and M2 macrophages in *P. gingivalis* W83 group were both higher than those in ΔPG0352 group, while the ratio of M1/M2 was higher in the ΔPG0352 group. Alveolar bone absorption was lower in ΔPG0352 group.

**Conclusion:**

Sialidase facilitates *P. gingivalis* immune evasion by reducing M1 polarization, antigen presentation, and phagocytosis of infected macrophages.

## Introduction

Periodontitis is a chronic inflammatory disease caused by microorganisms, leading to the inflammation of the gums, alveolar bone resorption, and tooth loss (Gheisary et al., 2022). It is the sixth most prevalent disease in the world and has a significant effect on public health (Kassebaum et al., 2014). Periodontitis not only negatively affects chewing function and aesthetics but is also associated with a range of systemic diseases including diabetes, cardiovascular diseases, rheumatoid arthritis, and cancer (Lundberg et al., 2010; Whitmore and Lamont, 2014; Sanz et al., 2018; Sanz et al., 2020). The progression of periodontitis is mediated by the interaction between host aberrant immune response and putative periodontal pathogens (Jia et al., 2019). The gram-negative anaerobic bacterium, *Porphyromonas gingivalis* (*P. gingivalis*) is considered to be one of the most important periodontal pathogens(Morandini et al., 2014).

Macrophages are recognized as critical immune cells involved in the periodontal defense system and play crucial roles in the progression of periodontitis (Sima and Glogauer, 2013). Macrophages can be polarized into differential phenotypes, including classical activation (M1) and alternative activation (M2), responding to micro-environmental signals (Mosser, 2003). M1 macrophages produce pro-inflammatory cytokines, such as interleukin (IL)-12 and induced nitric oxide synthase (iNOS) and express CD80 and CD86; they also have the biological functions of scavenging pathogenic bacteria and exerting antigen-presenting function (Wilson, 2014; Shapouri-Moghaddam et al., 2018). M2 macrophages mainly inhibit inflammation and secrete anti-inflammatory factors, such as IL-10 and arginase-1 (Arg-1) and express CD206 and CD163 (Liu et al., 2017). *P. gingivalis* has showed multiple ways to evade the killing of macrophages (Zheng et al., 2021). For example, *P. gingivalis* can modify lipid A to escape Toll-like receptor 4 (TLR4) recognition (Slocum et al., 2014) and activate complement-receptor-3 (CR3) on macrophages to facilitate its intra-cellular survive (Hajishengallis et al., 2013).

Sialic acids (SA) occupy the terminals of oligosaccharide chains involved in a wide range of biological processes, including cellular interactions and microbial attachment (Varki, 2007). *P. gingivalis* obtains free SA by exogenous decomposition, instead of synthesizing sialic acids endogenously. Sialidases are a critical glycosyl hydrolase that cleave terminal sialic acids from glycoconjugates (Von Itzstein et al., 1993), which are virulence factor of *P. gingivalis* that involved in the pathogenesis of periodontitis. Sialidases interrupt the interaction between pathogens and host cells, leading to the host mistakenly recognizing the pathogens as host cells, which facilitates the invasion to the host and the immune response evasion (Muller, 1974; Amano et al., 2014; Smutova et al., 2014).

PG0352 is the sole gene that encodes sialidases in *P. gingivalis* W83. We have constructed a mutant strain (ΔPG0352) of *P. gingivalis* W83 lacking sialidase gene and its complement strain (comΔPG0352) and found that sialidase deficiency did not affect the growth of *P. gingivalis* W83, while the pathogenicity of ΔPG0352 was lower than that of *P. gingivalis* W83 (Li et al., 2012). Our previous study also showed that, compared with *P. gingivalis* W83, ΔPG0352 promoted higher expression level of IL-12 in M1 macrophages and was more easily cleared by macrophages (Yang et al., 2018). In this study, in order to explore the effects of sialidase in *P. gingivalis*, we infected macrophages with *P. gingivalis* W83, ΔPG0352, comΔPG0352, and *Escherichia coli* (*E. coli*). Macrophage were measured, and a rat periodontitis model was established to determine the M1 and M2 polarization of macrophages *in vivo*.

## Methods

### Bacterial culture

The construction of both ΔPG0352 and comΔPG0352 has been described previously (Li et al., 2017). *P. gingivalis* W83, ΔPG0352, and comΔPG0352 were cultured in trypticase soy broth (TSB) including vitamin K (1 μg/ml), hemin (5 μg/ml), and 5% defibrinated sheep blood at 37°C in anaerobic environment. *E. coli* DH5 α was purchased from Beyotime Biotechnology (Beyotime, Shanghai, China) and subsequently cultured in Luria-Bertani (LB) broth at 37°C. Bacteria were resuspended in RPMI 1640 media before incubation with macrophages.

### Cell culture and differentiation

Human monocytes U937 (ATCC CRL-1593.2) were purchased from the American Type Culture Collection (Manassas, VA, USA) and then cultured as previously described (Yang et al., 2018). Phorbol myristic acid (PMA, Sigma-Aldrich, Taufkirchen, Germany) 100 ng/ml was used to induce the differentiation of U937 into macrophages. After 48h, the PMA was removed, and the cells were fed with fresh medium without PMA. The cells were then cultured for 24h and used in subsequent studies.

### Transmission electron microscopy

Macrophages were infected with *P. gingivalis* W83, ΔPG0352, comΔPG0352 (MOI = 100), or *E. coli* (MOI = 1) (Chen et al., 2018) for 6h. Subsequently, the cells were collected and fixed in glutaraldehyde with 2.5% sodium dimethylarsenate for 1h. One hour later, the cells were washed with sterilized water and suspended in osmium solution (EM Sciences, Hatfield, PA, USA) for 1.5h, and then dehydrated in ethanol (30, 50, and 70%) and acetone solutions (80, 90, and 100%) for 30 min. Subsequently, they were embedded in Epox 812 resin (E.F. Fullam Inc., Latham, NY, USA). Ultrathin sections were cut with uranyl acetate and stained with lead citrate and were viewed using a transmission electron microscope (H-7650, HITACHI, Japan).

### Enzyme-linked immunosorbent assay and Griess reaction

Macrophages were infected with *P. gingivalis* W83, ΔPG0352, comΔPG0352 (MOI = 100), or *E. coli* (MOI = 1) for 24h. The levels of IL-12 and IL-10 in infected macrophages were measured using Enzyme-linked immunosorbent assay (ELISA) kits (CLOUD-CLONE, Wuhan, China) according to the manufacturer’s instructions. Additionally, NO production was measured using a Griess reaction (Beyotime, Shanghai, China), and optical density was measured using a microplate reader at 450 nm (InfiniteM200, TACON, Japan).

### Flow cytometry

Macrophages were infected with *P. gingivalis* W83, ΔPG0352, comΔPG0352 (MOI = 100), or *E. coli* (MOI = 1) for 24h. A Zombie Aqua™ Fixable Viability Kit (BioLegend, San Diego, California, USA) was used to determine cell viability. The viability of infected macrophages was over 88% for flow cytometry. Infected macrophages were suspended in cell staining buffer and were pre-incubated with Human TruStain FcX™ (BioLegend, San Diego, California, USA) for 5 min. Then, cells were incubated with CD68-APC (BioLegend, San Diego, California, USA), CD206-PE (BioLegend, San Diego, California, USA), CD80-FITC (BioLegend, San Diego, California, USA) or corresponding human IgG served as isotype control on ice for 20 min in the dark. The cells were run through a FACSCalibur flow cytometer (Becton Dickinson, Franklin Lakes, NJ, USA).

### Immunofluorescence

Infected macrophages were fixed with 4% paraformaldehyde for 20 min and treated with 0.5% Triton X-100 for 10 min. The macrophages were then blocked with 1% bovine serum albumin (Sigma-Aldrich, Taufkirchen, Germany) for 1h. Subsequently, they were incubated with a primary antibody against MHC-II (Abcam, Cambridge, MA, USA) at 4°C overnight, followed by a secondary antibody [1:50 dilution in phosphate buffered saline (PBS) solution] (Abcam, Cambridge, MA, USA) at 37°C for 1h. Cell membranes were visualized by staining with DiIC18 for 30 min. Finally, cell nuclei were counterstained with 4,6-diamidino-phenylindole (DAPI) for 5 min to observe cell nucleus. The cells were observed under a fluorescence microscope and fluorescence intensity was analyzed using National Institutes of Health (NIH) IMAGEJ analysis software.

### Phagocytosis assay

Macrophages were infected with *P. gingivalis* W83, ΔPG0352, comΔPG0352 (MOI = 100), or *E. coli* (MOI = 1) for 4h, which had been labeled with 0.1 mg/ml FITC (Sigma-Aldrich, Taufkirchen, Germany). Cells were washed in PBS to remove bacteria, which had not been phagocytosed. Then, the cells were run through a FACSCalibur flow cytometer (Becton Dickinson, Franklin Lakes, NJ, USA) to measure phagocytosis. The results were expressed as the percentage of macrophages containing bacteria (FITC^+^ cells) over total macrophages.

### Animals

Twenty-five adult Sprague Dawley rats (provided from Beijing Vital River Laboratory Animal Technology Co., Ltd., Beijing, China) with initial body weights of 220–250 g were used (Zhang et al., 2020). The sample size was determined based on “resource equation” method(Charan and Kantharia, 2013) (*E* = Total number of animals – Total number of groups, the adequate value of E between 10 and 20). All rats were acclimatized under specific pathogen-free conditions and were provided with regular chow and sterile water throughout the experiment. All experiments were approved by the experimental animal welfare and ethics committee of China medical university (Approval No. 2018105) and conducted according to the guidelines of National Institute of Health (NIH) in the USA regarding the care and use of animals for experimental procedures.

### Experimental periodontitis and groups

Before the experiment, kanamycin (1 mg/ml) was added to the drinking water for 7 days to inhibit endogenous bacteria that were not conducive to the colonization of *P. gingivalis*. They were randomly assigned to the following five groups: (A) Healthy Control: treated with PBS; (B) Ligation Control: treated with ligation and PBS; (C) *P. gingivalis* W83: treated with ligation and *P. gingivalis* W83; (D) ΔPG0352: treated with ligation and ΔPG0352; and (E) comΔPG0352: treated with ligation and comΔPG0352. To construct the periodontitis model, the rats were anesthetized with 30 mg/kg pentobarbital sodium, and the necks of both maxillary first molars were ligated with 0.2-mm diameter wires. Bacteria, including *P. gingivalis* W83, ΔPG0352, and comΔPG0352, were collected in the logarithmic growth stage (600 nm OD value of 0.8–1.0) and resuspended in 100 µl of PBS containing 2% carboxymethyl cellulose to 1×10^9^ CFU/ml, and inoculated in three experimental groups every 2 days. The control groups were inoculated with PBS containing 2% carboxymethyl cellulose (Sulijaya et al., 2019). After 8 weeks, these rats were sacrificed using an overdose of anesthetic. The left and right hemimaxillas were collected.

### Immunohistochemistry and immunofluorescence

The left hemimaxillas were decalcified in 10% ethylenediaminetetraacetic acid (EDTA) for 2 months. The samples were embedded in paraffin and were sliced into serial paraffin sections (5 μm) in the mesiodistal direction on the mandibular first molars. The sections were sealed at room temperature in 10% serum and 1% BSA for 2h. Subsequently, they were incubated with CD163 antibody (1:200) (Abcam, Cambridge, MA, USA) or iNOS antibody (1:100) (Abcam, Cambridge, MA, USA) at 4°C overnight, and then incubated with secondary antibody (1:20,000) (Abcam, Cambridge, MA, USA) at room temperature for 1h. After the DAB reaction, nuclei were counterstained with hematoxylin for 5 min. Finally, images were captured by microscope camera at 400× magnification. The positive cell counting was conducted by two examiners.

For the immunofluorescence assay, sections were incubated with (1:100) F4/80 (Affinity, Jiangsu, China) and iNOS (1:100) (Proteintech, Wuhan, China) or F4/80 (1:100) (Santa Cruz, Santa Cruz, California, USA) and CD206 (1:200) (Proteintech, Wuhan, China) at 4°C overnight, and then incubated with secondary antibody (1:20,000) (Abcam, Cambridge, MA, USA) for 1h. Cell nuclei were counterstained with DAPI. Samples were observed under a fluorescence microscope and analyzed using NIH IMAGEJ analysis software.

### Quantification of alveolar bone loss

The right was used to measure alveolar bone loss. Samples were stained with 1% methylene blue clearly to delineate the cemento-enamel junction (CEJ). In a blinded manner, two examiners (ZSW and CC) measured the distance from the alveolar bone crest (ABC) to the CEJ of the maxillary first molar on the mesial, central and distal of buccal and palatal sides. The mean value of six sites was calculated as the absorption of the alveolar bone of each tooth (Matsuda et al., 2016; Sulijaya et al., 2019). The amount of alveolar bone loss was measured from images using a stereoscopic microscope (DP2- BSW; Olympus, Tokyo, Japan).

### Statistical analysis

GraphPad Prism 9.0 was used for statistical analysis. The normality of data was analyzed by the Shapiro–Wilk test. Statistical differences between two groups were compared by the two-tailed Student’s test. Analysis of variance (ANOVA) was used to compare differences among multiple comparisons. Quantitative data was expressed as mean ± standard deviation, and *P* < 0.05 indicated a statistical difference.

## Results

### Macrophage morphology after bacterial infection

**Figure 1:**
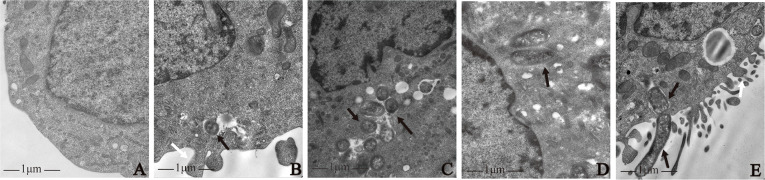
Morphological observation of macrophages infected with *P. gingivalis* W83, ΔPG0352, comΔPG0352, and *E*. *coli*. **(A)** Uninfected macrophages. Macrophages were infected with **(B)** *P. gingivalis* W83, **(C)** ΔPG0352, **(D)** comΔPG0352, and **(E)** *E*. *coli*. Black arrow: bacterial strains phagocytosed by macrophage; white arrows: pseudopods on the surface of macrophages; 25,000× magnification.

The results of transmission electron microscopy showed that all four strains induced macrophage phagocytosis. In the control group, macrophage membranes were intact, and macrophages were morphologically normal (**Figure 1A**). By contrast, on the surface of macrophages infected by the four strains, there were microvilli, cytoplasmic protrusions, and coated pits, and the membranes were discontinuous. Intra-cellular bacteria were enclosed in endocytic vacuoles. Macrophages extended pseudopodia to capture bacteria (**Figures 1B–E**).

### ΔPG0352 induced macrophages to release more M1-type inflammatory cytokines than *P. gingivalis* W83

**Figure 2:**
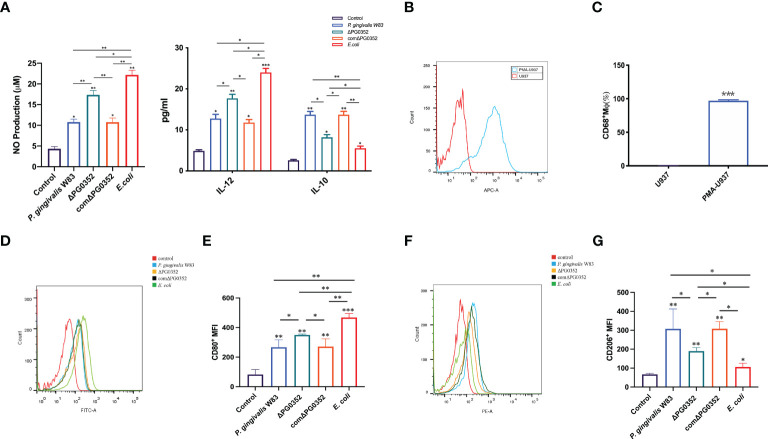
ΔPG0352 promoted M1 polarization of macrophage. **(A)** The levels of IL-12, iNOS, and IL-10. **(B, C)** The expression of M0 macrophages marker CD68. **(D, E)** The expression of M1 macrophage marker CD80. **(F, G)** The expression of M2 macrophage marker CD206. **(D, F)** *Y*-axis: number of cells; *X*-axis: relative intensity of fluorescence signal. Data are expressed as mean ± *SD* (*n* = 3), ^*^ *P* < 0.05, ^**^ *P* < 0.01, and ^***^ *P* < 0.001.

To clarify the effect of the four strains on macrophage polarization, we measured the levels of IL-12, iNOS and IL-10. Compared with the *P. gingivalis* W83 and comΔPG0352 groups, there were higher levels of IL-12 and iNOS expression in the ΔPG0352 and *E. coli* groups (*P* < 0.05), and the levels in the *E. coli* group were higher than those of the ΔPG0352 group (*P* < 0.05). The level of IL-10 was lower in the ΔPG0352 and *E. coli* groups than those in the *P. gingivalis* W83 and comΔPG0352 groups (*P* < 0.05), and the level in the *E. coli* group was lower than that of the ΔPG0352 group (*P* < 0.05). There were no significant differences between the *P. gingivalis* W83 and comΔPG0352 groups (*P* > 0.05) (**Figure 2A**).

### Compared with *P. gingivalis* W83, macrophages infected with ΔPG0352 express more M1 polarization surface markers

First, the level of macrophage surface markers CD68 was measured in order to detect M0 macrophages after PMA stimulation. We found that the expression level of CD68 on macrophages induced by PMA were 95.4%, while there was no CD68 expression on U937 cells without PMA stimulation (**Figures 2B, C**), suggesting that U937 differentiated into M0 macrophages after PMA inducing.

Next, we determined the polarization levels of infected macrophages by detecting M1 macrophages surface costimulatory molecule CD80 and M2 macrophages surface costimulatory molecule CD206. The results demonstrated that the levels of CD80 and CD206 in *P. gingivalis* W83, ΔPG0352, comΔPG0352, or *E. coli* groups were higher than those of the uninfected macrophages. Compared with macrophages infected by *P. gingivalis* W83, ΔPG0352-infected macrophages expressed higher level of CD80 and lower level of CD206, suggesting that ΔPG0352 promoted M1 polarization in macrophages more furtherly than *P. gingivalis* W83. *E. coli*–infected macrophages showed highest CD80 level and lowest CD206 level in all the infected ones (*P* < 0.05). There were no significant differences between the macrophages infected by *P. gingivalis* W83 and comΔPG0352 (*P* > 0.05) (**Figures 2D–G**).

### Macrophages infected with ΔPG0352 showed higher MHC-II expression and phagocytosis abilities

**Figure 3:**
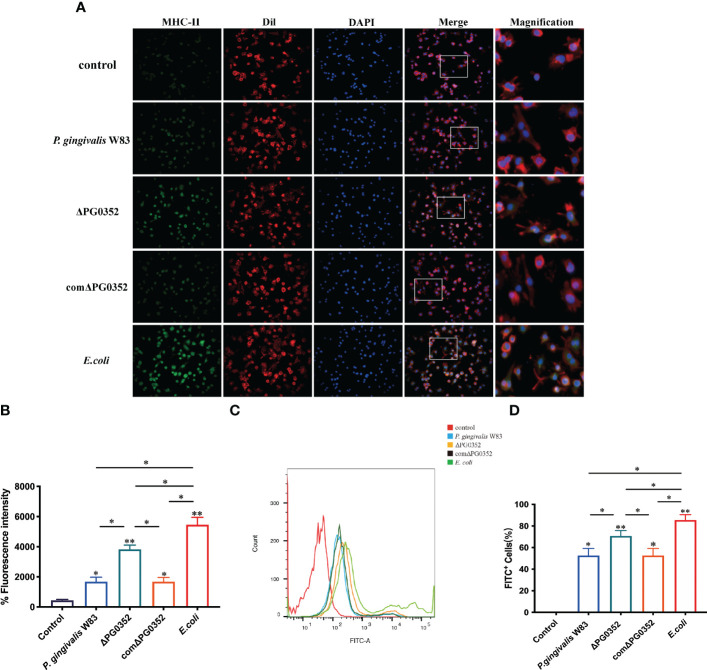
ΔPG0352 enhanced the expression of MHC-II and phagocytosis ability of macrophages. **(A)** The expression of MHC-II was determined by immunofluorescence. Green: MHC-II; red: cell membrane; blue: cell nucleus (400×). **(B)** The mean fluorescence intensity of MHC-II was calculated. **(C)** The phagocytosis ability of macrophages to *P. gingivalis* W83, ΔPG0352, comΔPG0352, and *E*. *coli* was assessed by flow cytometry. **(D)** FITC^+^ percentage of macrophages following *P. gingivalis* W83, ΔPG0352, comΔPG0352, and *E*. *coli*. Data are expressed as mean ± *SD* (*n* = 3), ^*^ *P* < 0.05 and ^**^ *P* < 0.01.

To determine the influence of sialidase deficiency on the MHC-II expression and phagocytosis in macrophages, immunofluorescence was performed to measure MHC-II expression level and flow cytometry was used to analyze phagocytosis of infected macrophages. Cell membranes and cell nucleus were stained to located the cells in the immunofluorescence assay. The fluorescence intensity of MHC-II in *P. gingivalis* W83, ΔPG0352, comΔPG0352, or *E. coli* groups were significantly higher than that in the control group. The fluorescence intensity of MHC-II in ΔPG0352-infected macrophages was higher than that in *P. gingivalis* W83-infected macrophages (*P* < 0.05). The fluorescence intensity of MHC-II was higher in the *E. coli–*infected macrophages than those in *P. gingivalis* W83, ΔPG0352, comΔPG0352-infected ones (*P* < 0.05). There were no significant differences between the *P. gingivalis* W83 and comΔPG0352 groups (*P* > 0.05) (**Figures 3A, B**). The results suggested that ΔPG0352 promoted the expression of MHC-II.

Flow cytometry results showed that the positive rate that macrophages phagocytosed *P. gingivalis* W83 was 59.5%. The positive rate in the ΔPG0352 group was 75.4%, and the *E. coli* group was 85.7%. There were no significant differences between the *P. gingivalis* W83 and comΔPG0352 groups (*P* > 0.05) (**Figures 3C, D**). These results suggested that more ΔPG0352 were phagocytosed by macrophages at the same time.

### Expression levels of iNOS and CD163 were higher in gingival tissues in the rat periodontitis model induced by *P. gingivalis* W83

**Figure 4:**
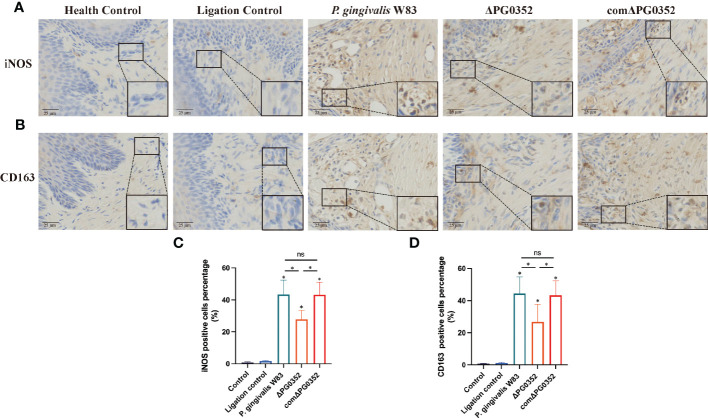
Expressions of iNOS and CD163 in the rat periodontitis model. **(A)** The expression of iNOS in gingival tissues from maxillary was assessed by immunohistochemistry at 400 × magnification. **(B)** The expression of CD163 in gingival tissues from maxillary was assessed by immunohistochemistry at 400 × magnification. **(C)** The positive cells percentage of iNOS (%). **(D)** The positive cells percentage of CD163 (%). ^ns^ No statistical significance, ^*^ *P* < 0.05.

Immunohistochemical results were shown in **Figure 4**. The positive expression densities of iNOS and CD163 in the gingival tissues were shown in **Figures 4C, D**. The levels of iNOS and CD163 in *P. gingivalis* W83, ΔPG0352, and comΔPG0352 groups were higher than those in health control and ligation control groups. The levels of iNOS and CD163 in *P. gingivalis* W83 and comΔPG0352 groups were higher than those in ΔPG0352 group (*P* < 0.05). These findings suggest that the expression levels of iNOS and CD163 were greater in gingival tissues in the *P. gingivalis* W83 group.

### The ratio of macrophage polarization M1 to M2 in periodontal tissues

**Figure 5:**
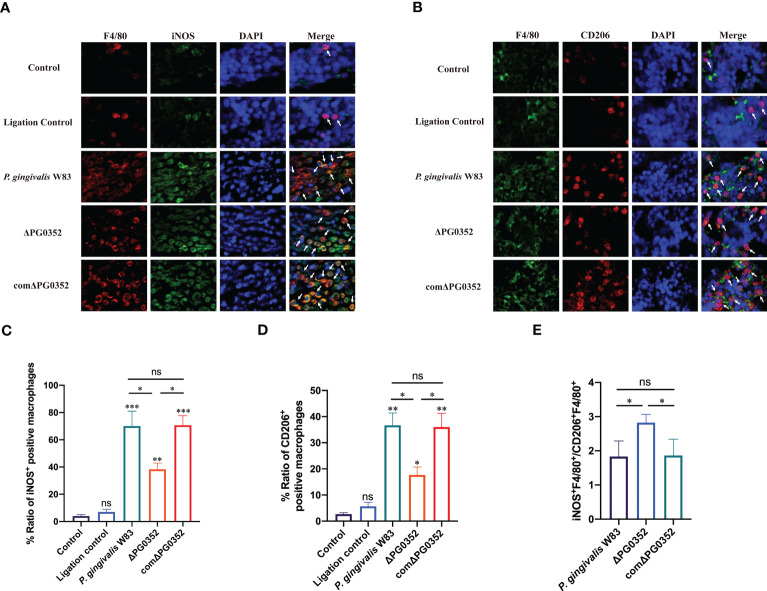
The ratio of M1 to M2 macrophages in the rat periodontitis model. **(A)** The expression of iNOS^+^F4/80^+^ in gingival tissues of rats. Red: macrophage; green: iNOS; blue: cell nucleus (400×). **(B)** The expression of CD206^+^F4/80^+^ in gingival tissues of rats. Green: macrophage; red: CD206; blue: cell nucleus (400×). **(C, D)** Percentage of iNOS^+^F4/80^+^ or CD206^+^F4/80^+^ macrophages. **(E)** The ratio of iNOS^+^F4/80^+^ to CD206^+^F4/80^+^ (M1/M2). Arrows indicate iNOS^+^F4/80^+^ or CD206^+^F4/80^+^ macrophages. Data are expressed as mean ± *SD* (*n* = 3), ^ns^ No statistical significance, ^*^ *P* < 0.05, ^**^ *P* < 0.01, and ^***^ *P* < 0.001.

To differentiate the phenotype of macrophages, we double-labeled macrophages with F4/80 and iNOS or F4/80 and CD206. The levels of iNOS^+^ F4/80^+^ and CD206^+^ F4/80^+^ in *P. gingivalis* W83 and comΔPG0352 groups were higher than those in ΔPG0352 group, and the expression levels of iNOS^+^ F4/80^+^ were higher than those of CD206^+^ F4/80^+^ in *P. gingivalis* W83, ΔPG0352, and comΔPG0352 groups (*P* < 0.05). However, compared with *P. gingivalis* W83 and comΔPG0352 groups, the ratio of iNOS^+^ F4/80^+^ to CD206^+^ F4/80^+^ (M1/M2) was 1.5-fold higher in ΔPG0352 group (*P* < 0.05) (**Figures 5A–E**). These findings suggest that, although levels of both iNOS^+^ F4/80^+^ and CD206^+^ F4/80^+^ were higher in the *P. gingivalis* W83 group, the ratio of iNOS^+^ F4/80^+^ to CD206^+^ F4/80^+^ (M1/M2) was higher in the ΔPG0352 group.

### Alveolar bone resorption

**Figure 6:**
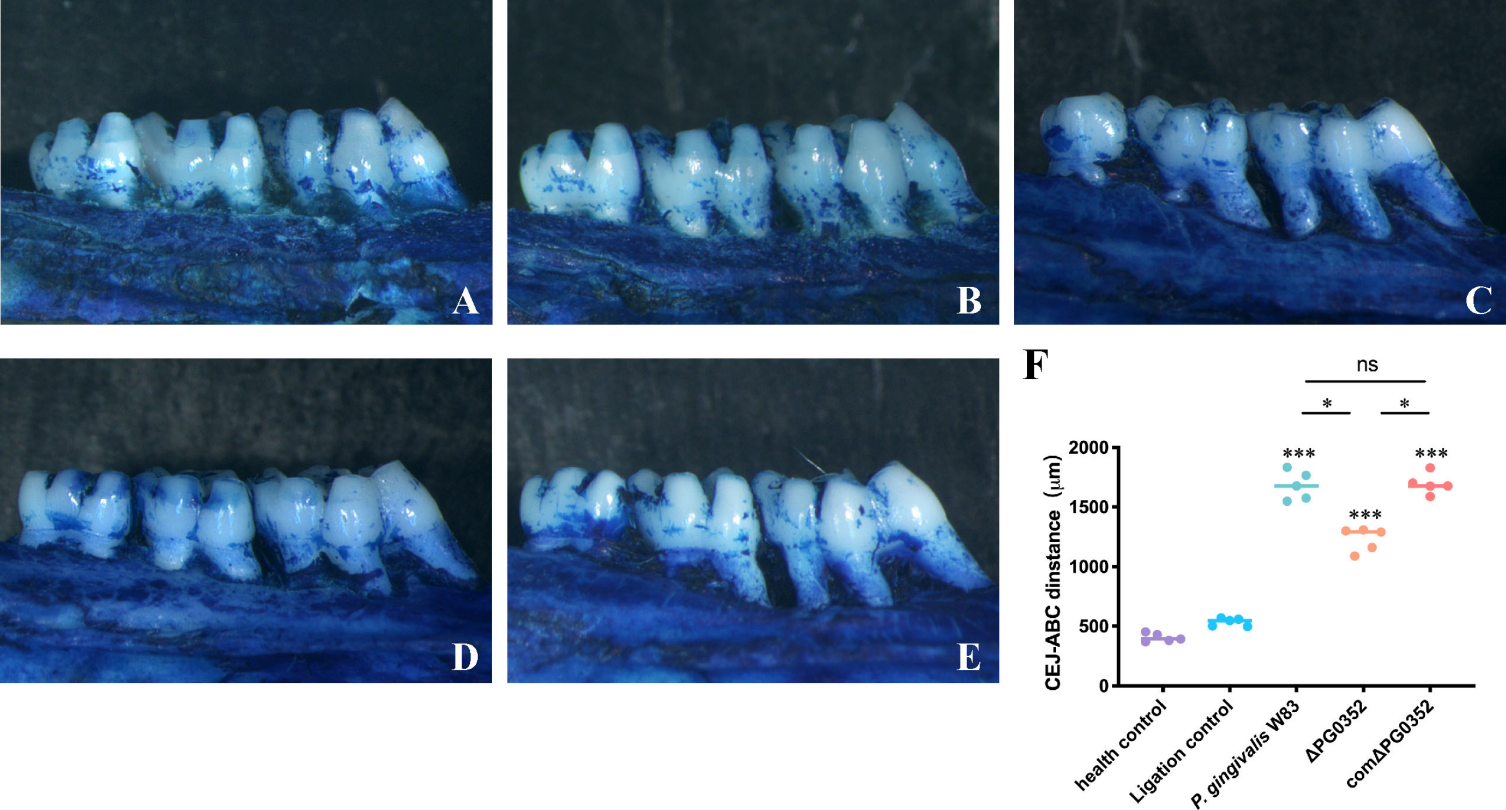
Alveolar bone resorption in the rat periodontitis model assessed by stereoscopic microscope. **(A)** Health control. **(B)** Ligation control. **(C)** *P. gingivalis* W83. **(D)** ΔPG0352. **(E)** comΔPG0352. **(F)** CEJ-ABC distance. Data are expressed as mean ± *SD* (*n* = 3), ^ns^ No statistical significance, ^***^ *P* < 0.001 compared with the control and ligation control groups, ^*^ *P* < 0.05, and 100× magnification.

We observed alveolar bone resorption in rats using stereoscopic microscopy (**Figure 6**). Health control group shows the position of the alveolar crest without alveolar bone resorption. Ligation control group shows mild alveolar bone resorption, while the alveolar bone in *P. gingivalis* W83, ΔPG0352, and comΔPG0352 groups showed obvious alveolar bone resorption. The absorption degree of alveolar bone in *P. gingivalis* W83 and comΔPG0352 groups was 1680.40 ± 121.37 μm and 1694.20 ± 86.46 μm, respectively and significantly higher than that in ΔPG0352 group (*P* < 0.05). These results suggest that ΔPG0352 induced lower alveolar bone resorption in the rat periodontitis model.

## Discussion

The occurrence and progression of periodontitis result from the interaction between periodontal pathogens and the host immune systems. *P. gingivalis* is one of the major etiologic agents of periodontitis. Macrophages involve in the resistance of exogenous microorganisms by polarization, antigen presentation, and phagocytosis. The results of transmission electron microscopy showed that all *P. gingivalis* W83, ΔPG0352, comΔPG0352, and *E. coli* induced macrophage phagocytosis. Microvilli formed on the surface of macrophages infected by bacteria. Macrophages phagocytosed these four strains and enclosed them in endocytic vacuoles within the cytoplasm. In addition, pseudopodia were observed in macrophages infected with *P. gingivalis* W83, ΔPG0352, *E. coli*, and comΔPG0352 in immunofluorescence assay. This indicated that *P. gingivalis* W83, ΔPG0352, and *E. coli* can activate macrophages. Activated macrophages usually show cells enlarged in size and increased lysosomal enzyme content and more active metabolism, and greater ability to kill intra-cellular pathogens (Franca et al., 2019). Such changes occur in M1 macrophages (Franca et al., 2019), the primary effector cells for killing microorganisms.

Many previous studies focused on the study of *P. gingivalis* or *E. coli* lipopolysaccharide (LPS) infection of macrophages. In this study, living bacteria was used because *P. gingivalis* expressed many virulence factors and some of these macro-molecules were modified by sialylation on the surface. Our results suggested that, compared with *P. gingivalis* W83, ΔPG0352 promoted M1 polarization more strongly. The reason may be that sialylation on the surface of bacteria affected the interaction between bacteria and cells, and host immune cells mistakenly recognized the bacteria as host cells, which inhibited the activation of immune cells, such as macrophage polarization. Sialidase deficiency in *P. gingivalis* W83 made it challenging to acquire sialic acid; thus, ΔPG0352 was recognized by macrophages more easily. In addition, lack of sialidase could reduce the activity of *P. gingivalis* fimbria and gingipain and reduce the layer of capsule that are often beneficial for *P. gingivalis* to evade immune clearance (Li et al., 2012). Gingipain could degrade chemotaxis and immunomodulation activity of some critical immune mediators such as interleukin-18 (IL-18) and tumor necrosis factor- *α* (TNF- *α*), thereby avoiding the killing by macrophages (Imamura, 2003). Encapsulated *P. gingivalis* could exhibit greater virulence than non-encapsulated *P. gingivalis* and evade host immune responses (Singh et al., 2011). Our previous study showed that the activity of gingipain of ΔPG0352 was lower than that of *P. gingivalis* W83 and ΔPG0352 failed to produce an intact capsule (Li et al., 2012). We also found that ΔPG0352 induced less expression of macrophage surface complement receptor 3 (CR3) than *P. gingivalis* W83 (Yang et al., 2018). The high expression of CR3 receptor could promote the activation of extracellular signal-regulated kinases ERK (1/2), and then reduce the expression of IL-12 and inhibit IL-12–mediated bacterial clearance (Trinchieri, 2003). Taken together, compared with *P. gingivalis* W83, the study suggested that ΔPG0352 promoted more macrophages M1 polarization.

Once inflammatory response in periodontal tissues was not be early controlled, antigen-presenting cells will process pathogens and present them to T cells and finally stimulate adaptive immune responses. We found that, compared with *P. gingivalis* W83 group, the expression levels of MHC-II and CD80 were higher, while the levels of CD206 was lower in the ΔPG0352 group. *P. gingivalis* can inhibit the surface expression of MHC-II on macrophages; thus, macrophages showed lower antigen presentation capacity. MHC-II downregulation on macrophages diminished CD4^+^ T cell responses (Milillo et al., 2019), contributing to immune escape of *P. gingivalis*. Our study suggested that, unlike *P. gingivalis* W83, ΔPG0352 abrogates the downregulation of MHC-II. Extracellular antigens are presented by macrophage through internalizing, processing, and loading the peptides onto MHC-II molecules, which are exposed on the cell membrane to activate CD4^+^ T cells with the assistance of the costimulatory molecules CD80 and CD86. In this study, the expression levels of both MHC-II and CD80 were higher on the ΔPG0352 group. We supposed that sialidase deficiency can abrogate the inhibition of *P. gingivalis* W83 on macrophages antigen presenting ability. In addition, the immune environment influences the response of macrophages on *P. gingivalis* (Papadopoulos et al., 2017). The inflammatory cytokine IL-12 could promote the expression of MHC-II and CD80 (Choi et al., 2019), while IL-10 inhibit the expression of MHC-II (Wu et al., 2018). In this study, comparing with *P. gingivalis* W83, ΔPG0352 promoted the expression of IL-12 and inhibited the expression of IL-10, which may be beneficial to promote the expression of MHC-II and CD80. Furthermore, *P. gingivalis* releases outer membrane vesicles into the surrounding environment (Srisatjaluk et al., 2002; Volgers et al., 2018). Outer membrane vesicles generally retain the complete outer membrane components of the bacterial cell wall, including protein, capsule, and fimbria (Srisatjaluk et al., 2002; Singhrao and Olsen, 2018). Outer membrane vesicles could inhibit MHC-II expression (Srisatjaluk et al., 2002). According to our previous study, lack of sialidase weakened the virulence of *P. gingivalis* W83 and reduced the activity of *P. gingivalis* W83 fimbria and capsule (Li et al., 2012), leading to changes in the composition or structure of the outer membrane vesicles (Helander et al., 1998; Ernst et al., 2001; Kong et al., 2011). As a result, there was a modulation of the activity of outer membrane vesicles on ΔPG0352 and the reversal of the inhibition of the outer membrane vesicles on MHC-II expression. In this study, we showed that ΔPG0352 was easier to be phagocytosed by macrophages. Certain virulence factors of *P. gingivalis* W83 such as capsule and gingipain, can help it escape phagocytosis by macrophages (Singh et al., 2011; Castro et al., 2017). The activities of capsule and gingipain on ΔPG0352 were reduced, leading to promotion of phagocytosis.

To further investigate the effect of *P. gingivalis* sialidase on macrophages *in vivo*, we established a rat periodontitis model and found that the expression level of iNOS^+^F4/80^+^ (M1 macrophage) was higher than those of CD206^+^F4/80^+^ (M2 macrophage) in each group. Compared with *P. gingivalis* W83, the expression levels of iNOS^+^F4/80^+^ and CD206^+^F4/80^+^ were lower in the ΔPG0352 group. It is noteworthy that the ratio of iNOS^+^F4/80^+^ to CD206^+^F4/80^+^ (M1/M2) was higher in ΔPG0352. It is possible that *P. gingivalis* W83 induced a more serious inflammatory response, and ΔPG0352 induced a relatively slight inflammatory response but promoted more macrophages to M1 polarization. We showed that the resorption of alveolar bone in the ΔPG0352 group was lower than that of the *P. gingivalis* W83 group. The result *in vitro* showed that, compared with *P. gingivalis* W83, ΔPG0352 was easier to be phagocytosed by macrophages. ΔPG0352 increased higher MHC-II expression levels, which enhanced macrophage antigen presentation ability and activated the host adaptive immune response, while *P. gingivalis* W83 tended to evade immune clearance. Consequently, it continuously colonized periodontal tissues and aggravated the inflammation of the periodontal tissues. In addition, compared with ΔPG0352, *P. gingivalis* W83 induced higher expression of iNOS^+^F4/80^+^ macrophages. iNOS could stimulate alveolar bone loss and plays an important role in the pathogenesis of periodontitis (Herrera et al., 2011).

This study suggested that, compared with *P. gingivalis* W83, DPG0352 promoted the polarization, enhanced antigen presentation, and was easier to be phagocytosed by macrophages. Lower resorption of alveolar bone was showed in the ΔPG0352 group in the rat periodontitis model. In summary, we demonstrated the importance of inhibiting the activity of sialidase in preventing progression of periodontitis.

## Data availability statement

The original contributions presented in the study are included in the article/supplementary material. Further inquiries can be directed to the corresponding authors.

## Ethics statement

The experimental animal welfare and ethics committee of China medical university (Approval No. 2018105) approved all experiments.

## Author contributions

XF was responsible for the conduction and manuscript writing of this study. SZ were responsible for conducting experiments and analyze the data. CC and LL were responsible for data curation. YS and HW contributed to were responsible for the conception of this study. YP contributed to the conception of this study. CL was responsible for the conception, manuscript writing and funding acquisition of this study. All authors contributed to the article and approved the submitted version.

## References

[B1] Amano A. Chen C. Honma K. Li C. Settem R. P. Sharma A. (2014). Genetic characteristics and pathogenic mechanisms of periodontal pathogens. Adv. Dent. Res. 26, 15–22. doi: 10.1177/0022034514526237 24736700 PMC6636228

[B2] Castro S. A. Collighan R. Lambert P. A. Dias I. H. Chauhan P. Bland C. E.. (2017). Porphyromonas gingivalis gingipains cause defective macrophage migration towards apoptotic cells and inhibit phagocytosis of primary apoptotic neutrophils. Cell Death Dis. 8, e2644. doi: 10.1038/cddis.2016.481 28252646 PMC5386511

[B3] Charan J. Kantharia N. D. (2013). How to calculate sample size in animal studies? J. Pharmacol. Pharmacother. 4, 303–306. doi: 10.4103/0976-500X.119726 24250214 PMC3826013

[B4] Chen S. Lu Z. Wang F. Wang Y. (2018). Cathelicidin-WA polarizes e. coli K88-induced M1 macrophage to M2-like macrophage in RAW264.7 cells. Int. Immunopharmacol. 54, 52–59. doi: 10.1016/j.intimp.2017.10.013 29101873

[B5] Choi J. N. Sun E. G. Cho S. H. (2019). IL-12 enhances immune response by modulation of myeloid derived suppressor cells in tumor microenvironment. Chonnam 55, 31–39. doi: 10.4068/cmj.2019.55.1.31 PMC6351325 30740338

[B6] Ernst R. K. Guina T. Miller S. I. (2001). Salmonella typhimurium outer membrane remodeling: role in resistance to host innate immunity. Microbes Infect. 3, 1327–1334. doi: 10.1016/S1286-4579(01)01494-0 11755422

[B7] Franca G. M. Carmo A. F. D. Costa Neto H. Andrade A. Lima K. C. Galvao H. C. (2019). Macrophages subpopulations in chronic periapical lesions according to clinical and morphological aspects. Braz. Oral. Res. 33, e047. doi: 10.1590/1807-3107bor-2019.vol33.0047 31141038

[B8] Gheisary Z. Mahmood R. Harri Shivanantham A. Liu J. Lieffers J. R. L. Papagerakis P.. (2022). The clinical, microbiological, and immunological effects of probiotic supplementation on prevention and treatment of periodontal diseases: a systematic review and meta-analysis. Nutrients 14. doi: 10.3390/nu14051036 PMC8912513 35268009

[B9] Hajishengallis G. Mcintosh M. L. Nishiyama S. I. Yoshimura F. (2013). Mechanism and implications of CXCR4-mediated integrin activation by porphyromonas gingivalis. Mol. Oral. Microbiol. 28, 239–249. doi: 10.1111/omi.12021 23331495 PMC4123224

[B10] Helander I. M. Latva-Kala K. Lounatmaa K. (1998). Permeabilizing action of polyethyleneimine on salmonella typhimurium involves disruption of the outer membrane and interactions with lipopolysaccharide. Microbiology 144 (Pt 2), 385–390. doi: 10.1099/00221287-144-2-385 9493375

[B11] Herrera B. S. Martins-Porto R. Maia-Dantas A. Campi P. Spolidorio L. C. Costa S. K.. (2011). iNOS-derived nitric oxide stimulates osteoclast activity and alveolar bone loss in ligature-induced periodontitis in rats. J. Periodontol. 82, 1608–1615. doi: 10.1902/jop.2011.100768 21417589 PMC3361509

[B12] Imamura T. (2003). The role of gingipains in the pathogenesis of periodontal disease. J. Periodontol. 74, 111–118. doi: 10.1902/jop.2003.74.1.111 12593605

[B13] Jia L. Han N. Du J. Guo L. Luo Z. Liu Y. (2019). Pathogenesis of important virulence factors of porphyromonas gingivalis *via* toll-like receptors. Front. Cell Infect. Microbiol. 9, 262. doi: 10.3389/fcimb.2019.00262 31380305 PMC6657652

[B14] Kassebaum N. J. Bernabe E. Dahiya M. Bhandari B. Murray C. J. Marcenes W. (2014). Global burden of severe periodontitis in 1990-2010: a systematic review and meta-regression. J. Dent. Res. 93, 1045–1053. doi: 10.1177/0022034514552491 25261053 PMC4293771

[B15] Kong Q. Yang J. Liu Q. Alamuri P. Roland K. L. Curtiss R. 3rd. (2011). Effect of deletion of genes involved in lipopolysaccharide core and O-antigen synthesis on virulence and immunogenicity of salmonella enterica serovar typhimurium. Infect. Immun. 79, 4227–4239. doi: 10.1128/IAI.05398-11 21768282 PMC3187260

[B16] Li C. Hu B. Bian J. Sun J. Zhang W. Liu J.. (2012). Abrogation of neuraminidase reduces biofilm formation, capsule biosynthesis, and virulence of porphyromonas gingivalis. Infect. Immun. 80, 3–13. doi: 10.1128/IAI.05773-11 22025518 PMC3255687

[B17] Li C. Yang X. Pan Y. Yu N. Xu X. Tong T.. (2017). A sialidase-deficient porphyromonas gingivalis mutant strain induces less interleukin-1beta and tumor necrosis factor-alpha in Epi4 cells than W83 strain through regulation of c-jun n-terminal kinase pathway. J. Periodontol. 88, e129–e139. doi: 10.1902/jop.2017.160815 28362225

[B18] Liu R. Fan T. Geng W. Chen Y. H. Ruan Q. Zhang C. (2017). Negative immune regulator TIPE2 promotes M2 macrophage differentiation through the activation of PI3K-AKT signaling pathway. PloS One 12, e0170666. doi: 10.1371/journal.pone.0170666 28122045 PMC5266285

[B19] Lundberg K. Wegner N. Yucel-Lindberg T. Venables P. J. (2010). Periodontitis in RA-the citrullinated enolase connection. Nat. Rev. Rheumatol 6, 727–730. doi: 10.1038/nrrheum.2010.139 20820197

[B20] Matsuda Y. Kato T. Takahashi N. Nakajima M. Arimatsu K. Minagawa T.. (2016). Ligature-induced periodontitis in mice induces elevated levels of circulating interleukin-6 but shows only weak effects on adipose and liver tissues. J. Periodontal. Res. 51, 639–646. doi: 10.1111/jre.12344 26667667

[B21] Milillo M. A. Trotta A. Serafino A. Marin Franco J. L. Marinho F. V. Alcain J.. (2019). Bacterial RNA contributes to the down-modulation of MHC-II expression on Monocytes/Macrophages diminishing CD4(+) T cell responses. Front. Immunol. 10, 2181. doi: 10.3389/fimmu.2019.02181 31572389 PMC6753364

[B22] Morandini A. C. Ramos-Junior E. S. Potempa J. Nguyen K. A. Oliveira A. C. Bellio M.. (2014). Porphyromonas gingivalis fimbriae dampen P2X7-dependent interleukin-1beta secretion. J. Innate Immun. 6, 831–845. doi: 10.1159/000363338 24925032 PMC4201861

[B23] Mosser D. M. (2003). The many faces of macrophage activation. J. Leukoc. Biol. 73, 209–212. doi: 10.1189/jlb.0602325 12554797

[B24] Muller H. E. (1974). Neuraminidase activity in streptococcus sanguis and in the viridans group, and occurrence of acylneuraminate lyase in viridans organisms isolated from patients with septicemia. Infect. Immun. 9, 323–328. doi: 10.1128/iai.9.2.323-328.1974 4816461 PMC414804

[B25] Papadopoulos G. Shaik-Dasthagirisaheb Y. B. Huang N. Viglianti G. A. Henderson A. J. Kantarci A.. (2017). Immunologic environment influences macrophage response to porphyromonas gingivalis. Mol. Oral. Microbiol. 32, 250–261. doi: 10.1111/omi.12168 27346827 PMC5192000

[B26] Sanz M. Ceriello A. Buysschaert M. Chapple I. Demmer R. T. Graziani F.. (2018). Scientific evidence on the links between periodontal diseases and diabetes: consensus report and guidelines of the joint workshop on periodontal diseases and diabetes by the international diabetes federation and the European federation of periodontology. J. Clin. Periodontol. 45, 138–149. doi: 10.1111/jcpe.12808 29280174

[B27] Sanz M. Marco Del Castillo A. Jepsen S. Gonzalez-Juanatey J. R. D'aiuto F. Bouchard P.. (2020). Periodontitis and cardiovascular diseases: consensus report. J. Clin. Periodontol. 47, 268–288. doi: 10.1111/jcpe.13189 32011025 PMC7027895

[B28] Shapouri-Moghaddam A. Mohammadian S. Vazini H. Taghadosi M. Esmaeili S. A. Mardani F.. (2018). Macrophage plasticity, polarization, and function in health and disease. J. Cell Physiol. 233, 6425–6440. doi: 10.1002/jcp.26429 PMC13482560 29319160

[B29] Sima C. Glogauer M. (2013). Macrophage subsets and osteoimmunology: tuning of the immunological recognition and effector systems that maintain alveolar bone. Periodontol 2000 63, 80–101. doi: 10.1111/prd.12032 23931056

[B30] Singh A. Wyant T. Anaya-Bergman C. Aduse-Opoku J. Brunner J. Laine M. L.. (2011). The capsule of porphyromonas gingivalis leads to a reduction in the host inflammatory response, evasion of phagocytosis, and increase in virulence. Infect. Immun. 79, 4533–4542. doi: 10.1128/IAI.05016-11 21911459 PMC3257911

[B31] Singhrao S. K. Olsen I. (2018). Are porphyromonas gingivalis outer membrane vesicles microbullets for sporadic alzheimer's disease manifestation? J. Alzheimers Dis. Rep. 2, 219–228. doi: 10.3233/ADR-180080 30599043 PMC6311351

[B32] Slocum C. Coats S. R. Hua N. Kramer C. Papadopoulos G. Weinberg E. O.. (2014). Distinct lipid a moieties contribute to pathogen-induced site-specific vascular inflammation. PloS Pathog. 10, e1004215. doi: 10.1371/journal.ppat.1004215 25010102 PMC4092147

[B33] Smutova V. Albohy A. Pan X. Korchagina E. Miyagi T. Bovin N.. (2014). Structural basis for substrate specificity of mammalian neuraminidases. PloS One 9, e106320. doi: 10.1371/journal.pone.0106320 25222608 PMC4164519

[B34] Srisatjaluk R. Kotwal G. J. Hunt L. A. Justus D. E. (2002). Modulation of gamma interferon-induced major histocompatibility complex class II gene expression by porphyromonas gingivalis membrane vesicles. Infect. Immun. 70, 1185–1192. doi: 10.1128/IAI.70.3.1185-1192.2002 11854199 PMC127778

[B35] Sulijaya B. Yamada-Hara M. Yokoji-Takeuchi M. Matsuda-Matsukawa Y. Yamazaki K. Matsugishi A.. (2019). Antimicrobial function of the polyunsaturated fatty acid KetoC in an experimental model of periodontitis. J. Periodontol. 90, 1470–1480. doi: 10.1002/JPER.19-0130 31343074

[B36] Trinchieri G. (2003). Interleukin-12 and the regulation of innate resistance and adaptive immunity. Nat. Rev. Immunol. 3, 133–146. doi: 10.1038/nri1001 12563297

[B37] Varki A. (2007). Glycan-based interactions involving vertebrate sialic-acid-recognizing proteins. Nature 446, 1023–1029. doi: 10.1038/nature05816 17460663

[B38] Volgers C. Savelkoul P. H. M. Stassen F. R. M. (2018). Gram-negative bacterial membrane vesicle release in response to the host-environment: different threats, same trick? Crit. Rev. Microbiol. 44, 258–273. doi: 10.1080/1040841X.2017.1353949 28741415

[B39] Von Itzstein M. Wu W. Y. Kok G. B. Pegg M. S. Dyason J. C. Jin B.. (1993). Rational design of potent sialidase-based inhibitors of influenza virus replication. Nature 363, 418–423. doi: 10.1038/363418a0 8502295

[B40] Whitmore S. E. Lamont R. J. (2014). Oral bacteria and cancer. PloS Pathog. 10, e1003933. doi: 10.1371/journal.ppat.1003933 24676390 PMC3968118

[B41] Wilson H. M. (2014). SOCS proteins in macrophage polarization and function. Front. Immunol. 5, 357. doi: 10.3389/fimmu.2014.00357 25120543 PMC4112788

[B42] Wu C. Liu C. Luo K. Li Y. Jiang J. Yan F. (2018). Changes in expression of the membrane receptors CD14, MHC-II, SR-a, and TLR4 in tissue-specific Monocytes/Macrophages following porphyromonas gingivalis-LPS stimulation. Inflammation 41, 418–431. doi: 10.1007/s10753-017-0698-y 29150769

[B43] Yang X. Pan Y. Xu X. Tong T. Yu S. Zhao Y.. (2018). Sialidase deficiency in porphyromonas gingivalis increases IL-12 secretion in stimulated macrophages through regulation of CR3, IncRNA GAS5 and miR-21. Front. Cell Infect. Microbiol. 8, 100. doi: 10.3389/fcimb.2018.00100 29675399 PMC5895773

[B44] Zhang J. Liu X. Wan C. Liu Y. Wang Y. Meng C.. (2020). NLRP3 inflammasome mediates M1 macrophage polarization and IL-1beta production in inflammatory root resorption. J. Clin. Periodontol. 47, 451–460. doi: 10.1111/jcpe.13258 31976565

[B45] Zheng S. Yu S. Fan X. Zhang Y. Sun Y. Lin L.. (2021). Porphyromonas gingivalis survival skills: immune evasion. J. Periodontal. Res. doi: 10.1111/jre.12915 34254681

